# Protocol for a systematic literature review and network meta-analysis of the evidence for therapies in allergic bronchopulmonary aspergillosis (ABPA)

**DOI:** 10.1186/s13643-026-03113-0

**Published:** 2026-03-11

**Authors:** Lisa Nwankwo, Ian Maidment, Jimstan Periselneris, David J. Jackson, Ritesh Agarwal, Koichiro Asano, Ali Nuh, Melody Ni, Anand Shah, Darius Armstrong-James

**Affiliations:** 1 Pharmacy Department, Royal Brompton and Harefield Hospital, Guy’s and St Thomas’ Hospital, London, SW3 6NP UK; 2 Aston Pharmacy School, College of Health and Life Science, College of Health and Life Sciences, Aston University, Birmingham, B4 7ET UK; 3 King’s College Hospital, Denmark Hill, London, SE5 9RS UK; 4 Guy’s Severe Asthma Centre, School of Immunology & Microbial Sciences, Guy’s Hospital, King’s College London, London, SE1 9RT UK; 5 Department of Pulmonary Medicine, Postgraduate Institute of Medical Education and Research, Chandigarh, India; 6 Division of Pulmonary Medicine, Department of Medicine, Tokai University School of Medicine, Kanagawa, Japan; 7 Department of Surgery and Cancer, NIHR London in Vitro Diagnostics Cooperative, St Mary’s Hospital, Imperial College London, London, W2 1NY UK; 8 Department of Respiratory Medicine, Royal Brompton and Harefield Hospitals, Guy’s and St., Thomas’ NHS Foundation Trust, London, SW3 6NP UK; 9 National Heart and Lung Institute, Imperial College London, London, SW7 2AZ UK; 10 Department of Infectious Diseases, MRC Centre for Molecular Bacteriology and Infection, Imperial College London, London, SW7 2AZ UK; 11 Microbiology Department, Royal Brompton and Harefield Hospital, Guy’s and St Thomas’ Hospital London, London, SW3 6NP UK

**Keywords:** Allergic bronchopulmonary aspergillosis, ABPA, Therapeutics, Treatment, Outcomes, Systematic review, Network meta-analysis, Antifungals, Biologics

## Abstract

**Introduction:**

Allergic bronchopulmonary aspergillosis (ABPA) affects millions worldwide, yet current treatment approaches remain suboptimal due to a one-size-fits-all model that fails to account for the significant heterogeneity across affected populations. While corticosteroids and itraconazole are commonly prescribed, there are associated risks and uncertainties regarding efficacy. Additionally, the efficacy of alternatives such as newer and higher activity triazole antifungals, or the place of “biologic” therapies like anti-IL5 antagonists, has not been thoroughly investigated.

**Objectives:**

This review will assess treatment outcomes in patients with ABPA (Population) receiving antifungal or biologic therapies (Intervention) compared with corticosteroids, placebo, or alternative active treatments (Comparator), evaluating lung function (FEV₁), serological markers, exacerbation frequency, patient-reported outcomes, steroid-sparing effects, and adverse events (Outcomes) through a systematic review and network meta-analysis of published peer-reviewed studies.

**Inclusion criteria:**

All articles using the Population, Exposure/Intervention, Comparator, Outcomes, Duration, and Results (PECODR) framework related to human patients published in English from inception to the date of undertaking the study (present) will be included.

**Materials and methods:**

The following literature databases will be searched: MEDLINE, EMBASE, Cochrane Library Trials database, PubMed Central, Web of Science, and Scopus from inception to the present in collaboration with an experienced librarian. The primary researcher will screen the titles and abstracts; deduplication will be performed using Covidence®, and inclusion will be validated by a second checker. Quantitative analyses will be performed to summarise the results as means, frequency tables, and odds ratios of outcomes. Where there is sufficient data, a network meta-analysis will be conducted. The systematic review and network meta-analysis will be undertaken according to the Preferred Reporting Items for Systematic Reviews and Meta-analyses for Network Meta-analysis (PRISMA-NMA) framework.

**Systematic review registration:**

PROSPERO CRD42024443073.

**Supplementary Information:**

The online version contains supplementary material available at 10.1186/s13643-026-03113-0.

## Strengths and limitations of this study

- The proposed systematic literature review and network meta-analysis aims to assess randomized controlled trials as well as real-world evidence to establish current evidence-based treatment practices applicable to a diverse population of ABPA patients.
- By implementing a ranking system in a network for all treatment comparisons, clinicians will be able to evaluate each intervention based on their clinical benefits relative to potential risks.
- Combining randomized controlled trials with other study designs in a unified network meta-analysis may present challenges.

## Introduction

Allergic bronchopulmonary aspergillosis (ABPA) is an allergic lung disease caused by a hypersensitivity reaction to the antigens of Aspergillus fumigatus, which colonizes the airways of individuals with asthma, cystic fibrosis (CF), and other lung diseases [1]. Patients commonly present with difficult-to-treat asthma, recurrent pulmonary opacities, and bronchiectasis. The disease course of ABPA is characterized by frequent exacerbations related to asthma, bronchiectasis, and ABPA [2], which can lead to a significant decline in lung function. It additionally imposes a heavy burden on Health Services due to frequent hospital admissions and high treatment costs. As a chronic condition, the disease also negatively impacts patients’ mental health, relationships, finances, and overall quality of life [3].

ABPA is the most common form of pulmonary aspergillosis, affecting up to 4.8 million patients worldwide [4] and 178,000 (50,000–250,000) in the UK [5]. It is also notably prevalent among individuals with asthma. A recent systematic review estimated almost an 11.3% prevalence of ABPA in patients with asthma visiting tertiary care [6]. Despite the significant burden, almost all multicentre randomized controlled studies of antifungals for pulmonary aspergillosis have concentrated on invasive pulmonary aspergillosis, which is a much less common form [5]. The ABPA population is highly heterogeneous, encompassing individuals with bronchiectasis [7], asthma, or CF phenotypes. Despite this, the model of care for ABPA currently follows a one-size-fits-all approach; all patients are treated with corticosteroids to rapidly control symptoms, with many continuing on a low-dose maintenance regimen. Itraconazole is typically prescribed as the first-line antifungal [8–10], while biologics are considered off-label in patients who have not responded to antifungal therapy [11, 12].

There is a scarcity of studies on optimal therapeutic approaches in this setting. While oral corticosteroids are a mainstay for acute ABPA [13], evidence on the use of antifungals, particularly newer triazole antifungals, and “biologic” monoclonal antibodies, remains sparse. There are no randomized controlled trials (RCTs) for ABPA exacerbations following the acute phase, although current care models generally follow the approach for acute ABPA [9]. The efficacy of newer, more potent triazole antifungals like voriconazole, posaconazole, and isavuconazole remains underexplored. There are no head-to-head comparative studies or comprehensive epidemiological evaluations of the four triazoles or biologics in terms of efficacy and safety, especially in cardiorespiratory populations, where treatment is often prolonged and off-label. Real-world data on these therapies are also lacking.

Furthermore, while there are data to support the efficacy of itraconazole for ABPA, it is associated with CYP450-mediated drug interactions. Treatment failure rates may reach up to 40% [14], compounded by a high incidence of side effects [15]. These side effects are a major concern for patients, contributing to treatment discontinuation, particularly in chronic disease management [16]. Addressing these issues from the patient’s perspective is essential for improving long-term outcomes.

Biologic therapies, such as anti-IgE, anti-IL5, and anti-IL5R antagonists, are becoming increasingly common in managing asthma phenotypes [17]. However, their role alongside antifungals has not been thoroughly investigated. Currently, they are primarily used to help patients avoid systemic steroids and their associated side effects, typically in salvage settings where corticosteroids or antifungals have been ineffective, symptoms continue to progress, or antifungal treatments are not tolerated. There is an urgent need for effective therapies that can rapidly control disease activity and reduce the reliance on corticosteroids, given the well-documented risks associated with long-term steroid use.

Several systematic reviews and meta-analyses have recently examined the role of antifungals and biologics in ABPA, but important knowledge gaps persist. For example, a 2024 meta-analysis assessed five biologics for ABPA in asthma and CF, focusing on outcomes such as FEV1, exacerbations, eosinophils, total IgE, and computed tomography (CT) changes. However, it did not include analysis of other ABPA response markers such as galactomannan (GM) or Aspergillus-specific IgG/IgE, health-related quality-of-life measures, or other active therapy comparators [18]. The study supported the use of Omalizumab as a steroid-sparing treatment and for reducing exacerbations in ABPA, but there was high variability between studies. A 2023 review of nebulised amphotericin B for ABPA deemed that from the low-quality evidence, over half of patients remained exacerbation-free at 2 years, but no significant difference was seen versus controls, and the review did not compare NAB to other active treatments [19]. Other reviews remain confined to single agents such as omalizumab (PMID: 36581073) or exclusively on populations with CF [20–23]. Importantly, none have included network meta-analysis or offered head-to-head comparisons across all antifungal and biologic options. Recent Cochrane and narrative reviews highlight similar limitations, particularly the lack of RCTs and limited reporting of long-term or patient-reported outcomes [20–23]. These gaps underscore the need for a more comprehensive synthesis that integrates all available treatments, including newer triazoles and biologics, across asthma and CF phenotypes.

In the literature, a variety of endpoints have been used to evaluate outcomes in ABPA, but understanding the most relevant measures of therapeutic success remains a significant challenge in the field [9], especially regarding monoclonal antibodies, which are used off-label. Prior research on antifungals has predominantly focused on markers such as serum Aspergillus and total IgE levels to gauge fungal activity. There are few studies reporting FEV1 as an outcome measure [24], and the impact on Aspergillus IgG has only been evaluated in a limited number of studies [25]. In the same manner, quality-of-life questionnaires (QoL) assessing patient perceptions are inconsistently used [26] and have even been discouraged in routine care by an expert panel [9] as they are deemed cumbersome, and none are specific to ABPA, meaning that surrogate questionnaires have to be relied on. However, the expert group concluded that QoL questionnaires may be appropriate in clinical trials.

Considering these methodological limitations, heterogeneity in outcome measures, and gaps in the comparative evidence, we propose to conduct a systematic review and network analysis to the following: (1) evaluate the efficacy and adverse outcomes of antifungals and monoclonal antibodies in treating allergic bronchopulmonary aspergillosis (ABPA); and (2) identify optimal endpoints for treatment response evaluation in ABPA. Additionally, we aim to map ABPA treatment research trends to highlight unaddressed gaps in the field.

## Methods

### Objectives

This review will be framed using the PECODR framework (Population, Exposure/Intervention, Comparator, Outcome, Duration, Results) [27], which incorporates the time factor into Duration and effect estimates, result consistency, and NMA treatment ranking into Results. The detailed application of PECODR to this study is outlined in the inclusion criteria section. Time points will include early (2 and 6 weeks), intermediate (3 and 6 months), long-term (12 months), and end-of-study follow-up.

### Primary objective

i. Assessment of the effects of antifungal or biologic treatments, compared with corticosteroids or placebo, on lung function (FEV₁) in adults with ABPA at study timepoints.

### Secondary objectives

ii. Assessment of treatment effectiveness based on secondary endpoints including Aspergillus IgG, Aspergillus IgE, total IgE, steroid-sparing effect, and exacerbation frequency (asthma, ABPA, bronchiectasis).
iii. Assessment of the proportion of studies reporting patient-reported outcome measures in ABPA.
iv. Assessment of the impacts of treatments on respiratory-related quality of life, as measured by the St. George’s Respiratory Questionnaire (SGRQ), the Asthma Control Questionnaire (ACQ), or the Bronchiectasis Impact Measure (BIM).
v. Assessment of the harms with each treatment including mortality, adverse drug reactions (ADRs), liver function abnormalities (ALT or bilirubin > 2–4xULN), disease progression due to inefficacy, and drug discontinuation for any reason including ADRs.

We will perform a systematic literature review with network meta-analysis of the literature from inception to date to summarise the evidence on the efficacy and adverse outcomes of antifungal agents and monoclonal antibodies for the treatment of ABPA using an integrated comparison. The goal of a network meta-analysis (NMA), also known as multiple treatment meta-analysis or mixed treatment comparison, is to combine the effect sizes of several studies evaluating different interventions or treatments.

Because the literature is expected to be sparse, real-world evidence and non-controlled studies will be included in this study using the framework described by Efthimiou et al. to combine randomised and non-randomised evidence in a network meta-analysis [28]. The systematic review will be undertaken according to the Preferred Reporting Items for Systematic Reviews and Meta-analyses for Network Meta-analysis (PRISMA-NMA) framework [29], and the protocol has been registered in the PROSPERO database.

### Literature searches

Search strategies will be developed by the primary researcher with support from the information scientist based on the PECODR framework (Table 1).

**Table 1:** Inclusion criteria

1. Population (P): Adults ≥ 18 years with ABPA
2. Exposure/Intervention (E/I):
Any active antifungal or biologic drug used:
Itraconazole
Voriconazole
Posaconazole
Isavuconazole
Amphotericin B (*fungizone* ®)
Caspofungin
Omalizumab
Mepolizumab
Dupilumab
Reslizumab
Benralizumab
Tezepelumab
3. Comparators (C):
Include: Comparative studies of any design that evaluate outcomes in at least two population groups, comparing:
– an antifungal agent with placebo, another antifungal agent, an oral corticosteroid, or a biologic agent; or
– a biologic agent with placebo, an oral corticosteroid, or an antifungal agent for the treatment of ABPA
4. Outcomes (O), see also endpoints (Table 3):
Include if one or more of:
- Endpoint/outcome measures of effectiveness/PROMs are present
- Endpoint/outcome measure of harm/failure/drug discontinuation/side effects are documented
- Time factor-treatment durations/time to event are documented
5. Duration (D): Long-term outcomes measured at approximately 2 weeks, 6 weeks, 3, 6, 12 months, and the end of study follow-up
6. Results (R): Effect estimates such as mean difference (MD), standardised mean difference (SMD), odds ratio (OR), risk ratio (RR), and hazard ratio (HR) will be used to compare outcomes (e.g., FEV₁) across interventions. Treatment consistency, comparative effectiveness, and ranking will be assessed through network meta-analysis
7. Study design
Include: Retrospective/prospective observational studies, randomised controlled trials (RCT), reviews, cohort, cross-sectional, case controlled, case series, case reports. Studies:
-Must report original data
- Must include at least one extractable outcome
8. Language: Studies in English (all languages will be included and will aim to find a translation). For logistical purposes (e.g. no access to translation tools), if no translation can be found, it will be eliminated from the search
9. Other filters: Human studies only

*Key*: *ABPA* Allergic bronchopulmonary aspergillosis, *PROM* Patient reported outcome measure

The search strategies for MEDLINE and EMBASE will be developed using controlled vocabulary (MeSH and Emtree terms, respectively) alongside free-text keywords. Terms were mapped to the PECODR framework to ensure conceptual alignment with the review objectives.

The draft MEDLINE search strategy is provided in Appendix 1, with PECODR–to–search term mapping tables shown in Appendix 2 (MEDLINE) and Appendix 3 (EMBASE) to support reproducibility.

We will search the following literature databases, from inception to the present:

- EMBASE
- MEDLINE
- Cochrane Library Trials database
- PubMed Central
- Web of Science (Core collections)
- Scopus

Internet searches

- Google Scholar

Supplemental searches

Citation chasing will be used to identify potentially relevant studies. If the full text of a publication is not available through standard retrieval methods, the corresponding author will be contacted directly.

Clinical trial registries including ClinicalTrials.gov and equivalents, as well as government department websites, will also be searched to identify relevant ongoing or unpublished studies.

Unpublished or grey literature will not be searched as this review focuses on peer-reviewed academic evidence.

### Eligibility criteria

We outline our inclusion and exclusion criteria below, designed to capture relevant studies while minimizing the risk of overlooking pertinent information for our review objectives. A study will be included if it meets all the inclusion criteria and none of the exclusion criteria.

To ensure diagnostic consistency across included studies, we will apply the 2024 revised ISHAM-ABPA working group guidelines [9], alongside earlier 2013 criteria [30] as the diagnostic reference standard. These criteria incorporate clinical, radiological, and immunological parameters, providing a validated framework to distinguish ABPA from other forms of aspergillosis or from fungal sensitization (Table 2).

**Table 2:** Exclusion criteria

1. Population:
- Studies reporting paediatric data (< 18 years)
-Non-ABPA and other forms of aspergillosis
2. Exposure/Intervention: No treatment strategy
3. Comparators
Exclude: Studies with no data on relevant pathogen or treatment outcome criteria or appropriate comparator
4. Outcomes: Outcomes not listed in Table 3
5. Duration:
-Studies not reporting any follow-up timepoints
- Studies with only baseline data and no post-intervention outcomes
- Studies where duration of treatment or follow-up is unclear or not specified
6. Results:
- Studies that do not report any effect estimates for treatment outcomes (e.g., no mean or median values, no risk or odds ratios, no change from baseline)
- Studies that report only narrative findings without any extractable data
- Studies lacking sufficient data to calculate effect sizes or include in meta-analysis (e.g., missing sample size, missing standard deviation, event counts or themes)
7. Study design
Exclude: Guidelines, editorials, non-peer-reviewed materials, surveillance reports/conference abstracts
8. Language: Not in English AND where no translation could be found
9. Other filters:
- Studies reporting on non-human data (e.g. animals, plants)
- Studies reporting on non-fungal data (e.g. bacteria)

### Inclusion criteria

The inclusion criteria are shown in Table 1.

### Exclusion criteria

The exclusion criteria are shown in Table 2.

### Outcome measures

The outcome measures are shown in Table 3.

**Table 3:** Outcome measures

		Endpoints
Primary	Lung function	FEV_1_, FEV_1_% predicted, FVC, FVC % Predicted, FEV_1_/FVC ratio, FeNO, TLCO% predicted
Secondary	All exacerbation (asthma and ABPA)	◦ Annualized rate
	ABPA exacerbation	◦ Annualized rate of exacerbations treated with at least two weeks of glucocorticoids or treated with a course of antifungals, frequency, number, time factor ◦ Rescue medication use
	*Aspergillus* serological markers	
	Composite response	◦ Cough, dyspnoea
	Fungal diagnostics	◦ BDG, PCR, Aspergillus galactomannan index value
	Mortality	
	Adverse events	
	Antifungal or biologics discontinuation rate	◦ Discontinuation due to progression ◦ Withdrawals/Discontinuation due to adverse effects ◦ Discontinuation due to any other reason
	Antifungals/Biologics	◦ Successful reduction or withdrawal of OCS
	Patient reported outcomes	◦ SGRQ total score ◦ BIM ◦ ACQ ◦ Proportion of studies reporting quality of life measures
Tertiary	Rate of hospitalization	Health economic related endpoints ◦ Length of hospital stay (days) ◦ Unplanned hospital admissions

*Key*:

*ACQ *Asthma Control Questionnaire

*BDG *1–3-β-d-Glucan

*BIM *Bronchiectasis Impact Measure

*FBC *full blood count

*FVC *Forced Vital Capacity

*FeNO* fractional exhaled nitric oxide

*FEV *Forced expiratory volume

*FEV*_*1*_ Forced expiratory volume in 1 s

*MIC *Minimum inhibitory concentration

*OCS *oral corticosteroid

*PCR *polymerase chain reaction

*SGRQ *St George's Respiratory Questionnaire

*TDM *Therapeutic Drug Monitoring

*TLCO *transfer factor for carbon monoxide

### Relevant types of study design

Only studies that specifically aim to demonstrate a cause-and-effect relationship between an intervention and its outcome will be included. Study designs that incorporate suitable comparators, such as before/after, control/treatment, different interventions, as well as studies that combine both types of comparisons will be included.

Literature reviews will not be included in the systematic review itself, but they will be used to identify additional relevant studies, if necessary, by referencing cited sources, and to provide context for the conclusions of the systematic review.

### Potential effect modifiers and sources of heterogeneity

Potential effect modifiers will be identified to better understand how effects vary across studies. Indeed, several factors, such as the study design, study location (e.g. associated antifungal use regulations, availability), and the antifungal under consideration, can all contribute to result heterogeneity. The primary researcher will extract information about potential effect modifiers from studies included in the full text screening. This information will be stored in a dedicated database. A non-exhaustive list of potential effect modifiers is provided below:

- Study design
- Comparator type
- Disease stage/type: serologic allergic bronchopulmonary aspergillosis (ABPA-S) / allergic bronchopulmonary aspergillosis with bronchiectasis (ABPA-B)
- Patient type (e.g. CF or asthma)
- Length of follow-up
- Treatment duration

Effect modifiers will be statistically assessed via network meta-regression if there are sufficient data.

## Data extraction strategy

### Search record database

Covidence® systematic review software will be used by the reviewers to screen studies and extract the relevant data in collaboration with an experienced librarian. The primary researcher will screen the titles and abstracts using the eligibility criteria, and deduplication will be performed. Any articles with unclear inclusion or exclusion status will be subject to a full-text review. The reasons for articles being excluded at screening or failing eligibility criteria will be recorded, along with the number of articles progressing to the data charting/data extraction phase. All inclusions will be validated by a second checker.

Data from all included studies will be extracted and documented in an Excel database using a predefined spreadsheet validated by the review team. These extracted data records will be made available as an additional supplementary file. Information extraction will adhere to the PECODR elements, with recorded outcomes including outcome means, sample sizes, and measures of variation such as standard deviation, standard error, and confidence intervals. This extracted information will support the assessment of antifungal and biologic treatment effects in ABPA patients.

To ensure accuracy in data extraction, two reviewers from the research team will independently extract information from a subset comprising 10% of all articles. Any discrepancies will be resolved through discussion between the two reviewers, with unresolved disagreements referred to the full review team for consensus. This process will ensure consistency in information extraction and interpretation across the review team.

### Data charting/extraction

The following fields/variables will be extracted:

- Article title
- Authors
- Journal (as a proxy for academic discipline or specialty)
- Year of publication
- Study setting (field or laboratory experiment)
- Study design
- Study duration
- Inclusion period
- Country
- Cohort–population (asthma, CF, intensive care, chronic obstructive pulmonary disease (COPD), haematological cancer, transplant, Coronavirus disease 2019 (COVID-19), influenza, diabetes)
- Patient demographics: age, gender
- Diagnosis and comorbidities
- Disease stage/type–ABPA–S/ABPA–B/unstated
- Antifungal-type, dose, cost, order in which antifungal therapies are used
- Antifungal side effects grade/severity, type
- Biologic treatment type, dose, cost, order in which biological therapies are used
- Treatment duration
- Adverse effects (AEs)—withdrawals or discontinuation from the study due to AEs
- Drug interactions
- Mortality rates at the end of the study
- Survival analysis
- Spirometry
- Pulmonary radiological signs: bronchiectasis, atelectasis, ground-glass, infiltrates, opacification, consolidation, etc.
- Radiological report for grading the stage of disease

**Table:** 

Nodules (0,1)	Cavitation (0,1)	Pleural thickening (0, 1, 2,3)	Bronchial wall thickening (0, 1, 2, 3)	Airway plugging (0,1)
- Fungal Diagnostics- (BDG, PCR, Aspergillus galactomannan index value on respiratory samples)
- Microscopy—fungal culture and sensitivities (MIC)
- Mention of antifungal resistance
- Aspergillus serological markers
- Symptom burden (clinical signs, symptoms)
- Therapeutic drug monitoring (TDM)
- Host inflammation (biochemistry, FBC)
- Concomitant medication including liver enzyme inhibitors and inducers
- Secondary/concomitant bacterial infection or viral infection
- Adjunctive corticosteroid use
- Exacerbation–annualized rate
- Hospitalization-bed days
- Notation of successful reduction or withdrawal of OCS
- Questionnaires used, and if used–SGRQ/ACQ/BIM
- Time to event outcomes
- Outcomes (see Table 3)
- Time factor associated with outcomes

If any studies are found to be ineligible for inclusion at any phase of the study, they will be excluded, and the number of exclusions at each stage (and overall) will be recorded.

## Results synthesis

### Data synthesis and presentation

**Fig. 1:**
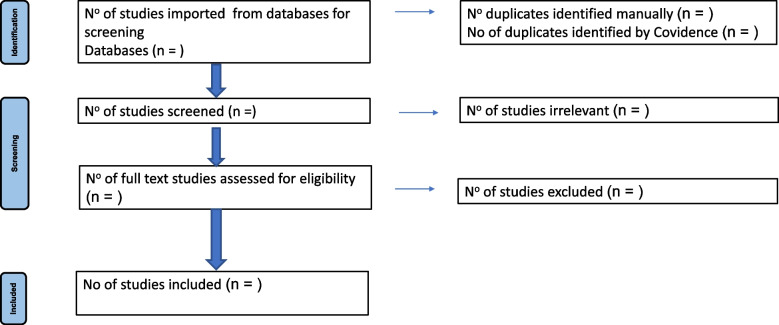
Planned PRISMA flow diagram for the systematic review of therapeutic outcomes in ABPA

In our analysis, we will construct frequency tables and cross-tabulations of results categorized by journal and year of publication. Graphs will be used to illustrate how the number of studies changed annually for each treatment, placebo, or no treatment. We will also compile a narrative report summarizing the occurrence and distribution of studies across regions, demographic groups, and knowledge disciplines. This will give a quantitative “map” of the key factors influencing the conduct of ABPA treatment research and offer deeper insight into research trends in this area from inception to the date of undertaking the study (present). The study selection process will be documented using a PRISMA 2020-compliant flow diagram [29], which will detail the number of records identified, screened, excluded (with reasons), and ultimately included. The diagram will be presented in the final review with a planned schema depicted in Fig. 1.

### Strategy for data synthesis

For each active therapy, the extracted data on the outcome criteria will be quantitatively analysed where sufficient data are available. Pooled estimates will only be reported where a minimum of three studies are available. Quantitative analysis will include descriptive statistics such as means and ranges for continuous outcomes, methods for exploring associations (e.g. correlation), and methods to compare groups (parametric and non-parametric tests for comparison of continuous or categorical data).

If data are insufficient for quantitative synthesis, results will be reported using a SWiM (synthesis without meta-analysis) approach [31]. Where adequate high-quality data exist, a network meta-analysis will be conducted.

### Risk-of-bias (quality) assessment

The risk of bias tool for randomized trials version 2 (ROB 2) [32] and the Risk of Bias Assessment Tool for Nonrandomized Studies 2 (RoBANS 2) tool [33, 34] will be used to evaluate the quality of the evidence. Each eligible study will be assessed across relevant domains; judgments of “low”, or “high” risk of bias will be made, and the risk–of–bias findings will be presented using visual summaries, including traffic–light (“red–amber–green”) plots. These visual outputs will clearly illustrate the distribution of bias assessments and support interpretation of the certainty of the evidence.

## Network meta-analysis

As a preliminary step, we will conduct pairwise meta-analyses of direct (head-to-head) comparisons where sufficient data exist. These analyses will serve as a benchmark for interpreting the network meta-analysis (NMA) findings and assessing consistency between direct and indirect evidence. Forest plots of these pairwise comparisons will be presented separately prior to constructing the network model, enabling transparency in effect estimation and potential heterogeneity in direct comparisons.

While traditional meta-analysis is useful, it can be limited in that it only compares two interventions at a time, and only those evaluated directly in head-to-head trials. Network meta-analysis (NMA) can be used to answer comparative effectiveness research questions when multiple interventions are available, and choices need to be made. Furthermore, network meta-analysis can estimate the relative rankings of interventions. In our study, multiple intervention groups, whether or not they have been directly compared in trials, will be analysed simultaneously using NMA based on assumptions of transitivity, similarity, and exchangeability of studies [35]. Since NMA is statistically complex, the analysis will be conducted in collaboration with a trained statistician.

### Defining the research question and treatment network

Two networks will be defined to answer the research question using the PECODR outlines:

What is the comparative effectiveness of antifungal treatments or biologics for improving outcomes (e.g. lung function) in patients with ABPA?

1. The first network will evaluate comparative treatment effect (outcomes in Table 3), and
2. The second will allow categorisation of therapies grouped by whether or not they affect Aspergillus IgG.

### Statistical analysis

The Bayesian NMA will be performed in a random-effects model using Markov chain Monte-Carlo methods [36] using the JAGS and the GeMTC package in R (https://gemtc.drugis.org/). This approach will allow for the reporting of overall effect sizes, as well as testing for heterogeneity, moderator effects, and publication bias.

The NMA will be carried out using a Bayesian hierarchical model rather than a frequentist model, because while the frequentist model produces binary outcomes with an arbitrary probability threshold of 0.5, the size of the effect can be difficult to understand. In contrast, a Bayesian approach allows prior knowledge from similar studies to be encoded into a statistical construct known as a prior, which is then combined with current experimental data to draw conclusions about the treatment under investigation. A random-effects meta-analysis will be employed, as it accounts for correlations in treatment effects at two levels: within-trial (probability level) and between-trial, making it useful for addressing heterogeneity among the results of multiple trials. Data will have been collected during the extraction phase of the systematic literature review, with additional data on potential effect modifiers extracted (link section) to evaluate transitivity [37].

### Conducting the network meta-analysis

In the Bayesian approach, a prior distribution of the parameters is needed to update the posterior distribution. The primary researcher will define a prior distribution that reflects existing beliefs about treatment efficacy outcomes in ABPA. This is unlikely to be an informative prior, as no larger network meta-analysis with the same context and treatments currently exists. Computational software such as the GeMTC package in R [36] automates most parts of the Bayesian inference process, including the selection of suitable prior distributions for all model parameters.

The primary researcher will assess the sensitivity of results to the choice of prior distribution [38]. This will include testing a range of priors, including non-informative and weakly informative priors, to assess the robustness of results to prior assumptions. The posterior distribution is a probability distribution that represents the updated beliefs about the parameter after incorporating the data. This will be generated using Markov Chain Monte Carlo (MCMC) simulation until convergence is achieved. The prior distribution will then be updated accordingly.

### Estimating effect

This will be demonstrated using a network diagram and network forest plots.

A network diagram is a graphical depiction of the structure of a network of interventions [39] which also serves to visually assess network geometry. Nodes represent the interventions, and lines show the available direct comparisons between pairs of interventions. In the multivariate random effects model, the reference treatment to which all treatments will be compared will be: placebo or no treatments or corticosteroid-only therapy or the most commonly used comparator. Network forest plots will be generated by pooling treatment effect size estimates (typically odds ratios or hazard ratios with corresponding 95% confidence intervals (CIs)).

Treatments will be ranked under the surfaces under the cumulative ranking curve (SUCRA) method [40]. SUCRA provides a percentage ranking (0–100%) for each intervention based on its likelihood of being among the most effective. Interventions with consistently high SUCRA values will be prioritised, but rankings will be interpreted cautiously alongside effect sizes, credible intervals, and the certainty of evidence.

Probability statements can then be made about the effectiveness of each treatment [41]. For example, for each treatment, the probability of being the best, second-best, or third-best among all treatments can be calculated. In interpreting the rankings, greater weight will be given to treatments that improve key clinical outcomes such as lung function and reduction in Aspergillus-specific serological markers, in line with standard therapeutic goals in ABPA.

### Running model diagnostics

The validity of the network meta-analysis is based on the fulfillment of the underlying assumptions [41].

#### Transitivity

The assumption of transitivity is that, other than the treatments being compared, there are no systematic differences between the comparisons in the network [39]. It assumes that the included studies are similar enough in terms of participant characteristics, interventions, and outcomes, allowing for indirect comparisons between treatments. Essentially, if A is better than B in one study and B is better than C in another, transitivity allows us to infer that A is likely better than C, even if there is no direct comparison between A and C.

Transitivity, as such, cannot be tested statistically, but the risk of violating this assumption can be reduced by including only studies in which the population, methodology, and target condition are as similar as possible [42]. By comparing population baseline characteristics across the included trials, we will evaluate whether there are significant differences that may affect the validity of combining the results. Additionally, epidemiological judgment is needed to assess whether the distribution of effect modifiers across studies permits reliable indirect comparisons [43, 44].

#### Assessing model validity

Inconsistency (i.e. discrepancy between direct and indirect comparisons); heterogeneity (i.e. clinical, methodological, and statistical variability within direct and indirect comparisons); and bias (among others) may influence effect estimates obtained from network meta-analyses.

##### Inconsistency

The statistical manifestation of transitivity is called consistency, and a lack thereof is known as inconsistency [43]. Consistency means that the relative effect estimate based on direct evidence does not differ from that based on indirect evidence [45]. Node splitting will be used to assess inconsistency [46]. If the node-splitting method reveals inconsistencies in some of the estimates, the researchers will re-check all included evidence for potential design differences. Additionally, global inconsistency across the entire network will be evaluated using Cochran’s Q test and quantified using the I2 statistic [43].

##### Heterogeneity

The restricted maximum likelihood method will be used to estimate heterogeneity. The approach suggested by Welton et al. [47] will also be used to include all data while simultaneously adjusting and down-weighting evidence from studies deemed to be at high risk of bias.

##### Similarity or exchangeability

Similarity (exchangeability) assumes that treatment effects are comparable across studies because patients, interventions, and outcome definitions do not differ in ways that modify the treatment effect. This will be assessed qualitatively by comparing trial-level covariates (e.g. age, disease severity, prior therapy, diagnostic criteria) across included studies to ensure there is no structural imbalance that could distort indirect comparisons.

##### Homogeneity in population and design

Homogeneity refers to the extent to which included studies are similar in design and population characteristics. Trials with excessive variation in design features (e.g. randomisation methods, follow-up duration, dose, or comparator choice) or population features (e.g. comorbidities, ABPA diagnostic thresholds) will be described and assessed for their influence on network stability through sensitivity analyses.

#### Assessing the convergence

Convergence will be evaluated using trace plots, Gelman–Rubin–Brooks plots and/or density plots of the posterior effect size estimates [48].

#### Assessing publication bias

Publication bias will be explored through visual inspection of funnel plot asymmetry for comparisons with ≥ 10 studies. Egger’s regression test will be used for continuous outcomes [49], and Harbord’s test for dichotomous data [50], where suitable. Comparison-adjusted funnel plots will also be generated to explore small-study effects in the network [51].

In addition, other potential biases specific to network meta-analysis, including small-study effects, selective outcome reporting, and imbalance in evidence contribution across comparisons, will be considered when interpreting results, in line with methodological guidance for network meta-analysis [43].

### Patient and public involvement

We currently have seven public and patient representatives sitting on our Research lay person advisory Group, who have already provided valuable input and advice regarding our planned systematic review. Being valued members of the research team, they will continue as co-collaborators, contributing essential patient perspectives informed by their lived experience.

## Discussion

Despite the clinical importance of allergic bronchopulmonary aspergillosis (ABPA), treatment remains largely empirical, with few robust comparative studies guiding therapeutic decisions. This systematic review and network meta-analysis aims to address this evidence gap by integrating data across antifungals and biologics, including real-world and non-randomized studies. The heterogeneity of ABPA phenotypes suggests that treatment response may vary significantly across populations. However, as no stratified approach currently informs treatment allocation, this review may highlight the need for phenotype-guided therapy. The absence of head-to-head comparisons of newer triazoles or biologics underscores the importance of this synthesis in generating comparative effectiveness data to support future guidelines and trial design.

Treatment response in ABPA is also likely influenced by comorbid conditions such as cystic fibrosis, asthma, or bronchiectasis, which differ in airway pathology and inflammatory drivers. These factors may shape response to corticosteroids, antifungals, or biologics, yet most existing studies do not stratify by underlying phenotype. Where possible, this review will explore differential treatment effects by comorbidity or baseline biomarkers, helping to identify gaps in subgroup-specific data and inform future personalised approaches.

A further challenge in evaluating treatment for ABPA is the diversity and inconsistency of outcome measures reported in the literature. While lung function indices such as FEV1 are objectively quantifiable, they are infrequently used as primary endpoints in existing studies. Serological markers (e.g. total IgE or Aspergillus-specific IgG) remain common but are not always reliable proxies for clinical improvement—particularly with biologic therapies that modulate host response rather than fungal burden directly. Similarly, patient-reported outcomes (PROs) are often excluded, underreported, or assessed using tools not validated for ABPA. This review will also evaluate the prevalence and quality of PRO reporting—an essential consideration given the chronic, symptom-driven nature of ABPA and the growing emphasis on patient-centered outcomes in respiratory research.

Finally, incorporating real-world evidence (RWE) and non-randomized studies poses both an opportunity and a methodological challenge. While RWE offers valuable insights into treatment durability, adherence, and safety outside tightly controlled trial settings, it increases susceptibility to bias and confounding. To address this, our methodology will adopt a structured framework for evidence synthesis that accounts for study design heterogeneity and applies rigorous risk-of-bias assessment. This pragmatic approach will reflect the current evidence landscape in ABPA and will allow us to deliver clinically useful comparative estimates while highlighting areas in urgent need of well-designed randomized controlled trials. Ultimately, we hope our systematic review will help provide recommendations to guide treatment guidelines.

## Supplementary Information

Additional file 1: Appendices.

## Data Availability

Not applicable.
