# Supplementary material for: Protocol for a systematic literature review and network meta-analysis of the evidence for therapies in allergic bronchopulmonary aspergillosis (ABPA)
Source: Syst Rev. 2026 Mar 11;15:140. doi: 10.1186/s13643-026-03113-0 (PMC13088818; doi:10.1186/s13643-026-03113-0)
Supplement: Supplementary file 1 — Additional file 1: Appendices. [file 13643_2026_3113_MOESM1_ESM.docx]

**Appendix 1. Draft MEDLINE Search Strategy**

("Allergic Bronchopulmonary Aspergillosis"[majr] OR "bronchopulmonary aspergillosis"[tiab] OR ABPA[tiab] OR "Aspergillus sensitisation"[tiab] OR "Aspergillus sensitization"[tiab])

AND

("Antifungal Agents"[majr] OR itraconazole[tiab] OR voriconazole[tiab] OR posaconazole[tiab] OR isavuconazole[tiab] OR "Amphotericin B"[tiab] OR caspofungin[tiab])

OR

("Monoclonal Antibodies"[majr] OR omalizumab[tiab] OR mepolizumab[tiab] OR dupilumab[tiab] OR reslizumab[tiab] OR benralizumab[tiab] OR tezepelumab[tiab])

AND

("Forced Expiratory Volume"[Mesh] OR FEV1[tiab] OR "Pulmonary Function"[tiab] OR "Lung function"[tiab] OR exacerbation*[tiab] OR "Asthma Exacerbation"[Mesh] OR "Immunoglobulin E"[Mesh] OR "Aspergillus IgG"[tiab] OR "Quality of Life"[Mesh] OR SGRQ[tiab] OR ACQ[tiab] OR BIM[tiab] OR "Drug Discontinuation"[tiab] OR "Adverse Effects"[tiab] OR mortality[tiab])

Filters: Humans[Mesh], English[lang], Adult[Mesh]

**Appendix 2: PECODR Mapping to Search Terms and MeSH Headings**

| **PECODR Element** | **Protocol Definition** | **Mapped Search Terms / MeSH Headings** |
| --- | --- | --- |
| **Population (P)** | Adults aged ≥18 years with ABPA | "Allergic Bronchopulmonary Aspergillosis"[majr] OR "bronchopulmonary aspergillosis"[tiab] OR ABPA[tiab] OR "Aspergillus sensitisation"[tiab] OR "Aspergillus sensitization"[tiab] |
| **Exposure/Intervention (E/I)** | Any active antifungal or biologic drug | "Antifungal Agents"[majr] OR itraconazole[tiab] OR voriconazole[tiab] OR posaconazole[tiab] OR isavuconazole[tiab] OR "Amphotericin B"[tiab] OR caspofungin[tiab]"Monoclonal Antibodies"[majr] OR omalizumab[tiab] OR mepolizumab[tiab] OR dupilumab[tiab] OR reslizumab[tiab] OR benralizumab[tiab] OR tezepelumab[tiab] |
| **Comparator (C)** | Placebo, corticosteroids alone, or other active drug | "Glucocorticoids"[Mesh], corticosteroids[tiab], prednisone[tiab], placebo[tiab], "standard of care"[tiab] (conceptually relevant, but not always directly searchable) |
| **Outcomes (O)** | FEV₁, exacerbation rate, IgE/IgG, PROMs, adverse events, mortality | "Forced Expiratory Volume"[Mesh], FEV1[tiab], "Pulmonary Function"[tiab], "Asthma Exacerbation"[Mesh], exacerbation*[tiab], "Immunoglobulin E"[Mesh], "Aspergillus IgG"[tiab], "Quality of Life"[Mesh], SGRQ[tiab], ACQ[tiab], BIM[tiab], "Drug Discontinuation"[tiab], "Adverse Effects"[tiab], mortality[tiab] |
| **Duration (D)** | Outcomes measured at 2w, 6w, 3m, 6m, 12m, or end of study | Will be captured during full-text data extraction |
| **Results (R)** | Effect estimates (e.g. MD, SMD), treatment ranking (NMA) | Will be captured during analysis/synthesis phase |
| **Study Design** | RCTs, observational studies, case series/reports | No study design filter applied at search level; design included at screening |
| **Language** | English language only | english[lang] |
| **Other Filters** | Human studies; adults only (≥18 years) | humans[Mesh] OR Humans[Filter], adult[MeSH] OR (adult*[tiab] OR "18 years"[tiab] OR "≥18"[tiab] OR "over 18"[tiab]) OR >18 years [Filter] |

**Key:**
MEDLINE terms:**[Mesh]** = Medical Subject Headings (controlled vocabulary); **[majr]** = Major MeSH topic; **[tiab]** = Title/Abstract fields (free-text search); **[lang]** = Limits by language (English); **[Filter]** = Built-in PubMed filters (e.g. Humans, Adults ≥18 years).

**Appendix 3. PECODR Mapping to Emtree Terms and Free Text (EMBASE)**

| **PECODR Element** | **Protocol Definition** | **Mapped Search Terms / Emtree Headings** |
| --- | --- | --- |
| **Population (P)** | Adults aged ≥18 years with ABPA | 'allergic bronchopulmonary aspergillosis'/exp OR "bronchopulmonary aspergillosis":ti,ab OR ABPA:ti,ab OR "aspergillus sensitisation":ti,ab OR "aspergillus sensitization":ti,ab |
| **Exposure/Intervention (E/I)** | Any active antifungal or biologic drug | 'antifungal agent'/exp OR itraconazole:ti,ab OR voriconazole:ti,ab OR posaconazole:ti,ab OR isavuconazole:ti,ab OR "amphotericin B":ti,ab OR caspofungin:ti,ab 'monoclonal antibody'/exp OR omalizumab:ti,ab OR mepolizumab:ti,ab OR dupilumab:ti,ab OR reslizumab:ti,ab OR benralizumab:ti,ab OR tezepelumab:ti,ab |
| **Comparator (C)** | Placebo, corticosteroids alone, or other active drug | 'glucocorticoid'/exp OR corticosteroid*:ti,ab OR prednisone:ti,ab OR placebo:ti,ab OR "standard of care":ti,ab |
| **Outcomes (O)** | FEV₁, exacerbation rate, IgE/IgG, PROMs, ADRs, mortality | 'forced expiratory volume'/exp OR FEV1:ti,ab OR "pulmonary function":ti,ab OR 'asthma exacerbation'/exp OR exacerbation*:ti,ab OR 'immunoglobulin E'/exp OR "Aspergillus IgG":ti,ab OR 'quality of life'/exp OR SGRQ:ti,ab OR ACQ:ti,ab OR BIM:ti,ab OR "drug discontinuation":ti,ab OR "adverse effects":ti,ab OR mortality:ti,ab |
| **Duration (D)** | 2w, 6w, 3m, 6m, 12m, end of study | Not directly searched — captured during data extraction |
| **Results (R)** | Effect estimates, NMA ranking | Not directly searched — handled in synthesis phase |
| **Study Design** | RCTs, observational studies, case series/reports | No study design filter applied at search level; applied during screening |
| **Language** | English only | AND [english]/lim |
| **Other Filters** | Human studies; adults ≥18 years | AND [humans]/lim AND [adult]/lim |

**Key:**

EMBASE terms:**/exp** = Exploded Emtree term (includes narrower related concepts); **:ti,ab** = Title and abstract fields (free-text search); **/lim** = Limits (e.g. [english]/lim restricts to English language); **/de** = Exact descriptor term in Emtree (no explosion); **[humans]/lim** = Limits to human studies; **[adult]/lim** = Limits to adult studies.
